# Exploring interdisciplinary perspectives on the implementation of *personalized medicine* and *patient-orchestrated care* in Alzheimer's disease: A qualitative study within the ABOARD research project

**DOI:** 10.1177/13872877251326166

**Published:** 2025-03-21

**Authors:** Tanja J de Rijke, Dianne Vasseur, Wiesje M van der Flier, Mirella MN Minkman, Hanneke FM Rhodius-Meester, Nicolaas A Verwey, Ellen MA Smets, Leonie NC Visser

**Affiliations:** 1 Amsterdam UMC, University of Amsterdam, Medical Psychology, Meibergdreef 9, Amsterdam, the Netherlands; 2 Alzheimercentrum Amsterdam, Neurologie, Vrije Universiteit Amsterdam, Amsterdam UMC, Amsterdam, the Netherlands; 3 Amsterdam Neuroscience, Neurodegeneration, Amsterdam, the Netherlands; 4 Amsterdam Public Health, Personalized Medicine & Quality of Care, Amsterdam, the Netherlands; 5 Vilans, the national Centre of expertise for care and support, Utrecht, the Netherlands; 6 Department of Epidemiology & Data Science, Vrije Universiteit Amsterdam UMC, Amsterdam, the Netherlands; 7 Tilburg University, TIAS School for business and society, Tilburg, the Netherlands; 8 Department of Internal medicine, Geriatric Medicine section, Vrije Universiteit Amsterdam, Amsterdam UMC, Amsterdam, the Netherlands; 9 Department of Geriatric Medicine, The Memory Clinic, Oslo University Hospital, Oslo, Norway; 10 Department of Neurology, Memory Clinic, Medical Center Leeuwarden, Leeuwarden, The Netherlands; 11 Division of Clinical Geriatrics, Center for Alzheimer Research, Department of Neurobiology, Care Sciences and Society, Karolinska Institutet, Stockholm, Sweden

**Keywords:** Alzheimer's disease, dementia, healthcare, implementation, interdisciplinary, interview, patient-centered care, policy, precision medicine, shared decision-making

## Abstract

**Background:**

The concepts of ‘*personalized medicine*’ and ‘*patient-orchestrated care*’ in Alzheimer's disease (AD) lack standard conceptualization, which presents challenges for collaborative and interdisciplinary care.

**Objective:**

We explored the interpretations and perspectives of professionals involved in interdisciplinary work on a large-scale project, “ABOARD”, with the aim to implement *personalized medicine* and *patient-orchestrated care* in AD.

**Methods:**

Semi-structured interviews were conducted with 30 professionals and audio-recorded. Two researchers independently coded the data inductively, followed by a thematic analysis.

**Results:**

According to professionals across different disciplinary backgrounds (mean age 45.7 years; 53.3% female), *personalized medicine* pertains to the relevant options that an individual has, informed by biomedical and psychosocial factors, whereas *patient-orchestrated care* captures factors relevant to the decision-making process. Professionals differed in their views on *patient-orchestrated care* regarding its desirability and feasibility. The concepts were viewed as similar by professionals, as both involve personal preferences while ultimately assigning responsibility to the clinician. However, implementation challenges persist, and no thematic differences were found between clinicians and other AD-related professionals.

**Conclusions:**

AD professionals have shared interpretations and perspectives on implementation of *personalized medicine* but differed in their views on patient-orchestrated care. Personal preferences are seen as part of *personalized medicine*, but not yet reflected in definitions in the AD field and beyond. Critical discussions on the challenges and existing doubts are necessary for both *personalized medicine* and *patient-orchestrated care*. Multi-level implementation changes are needed for both concepts, which warrants stakeholder involvement as well as support and resources from the entire AD field.

## Introduction

Alzheimer's disease (AD) is one of the major health problems of this century. Currently, 55 million people worldwide are affected by dementia, with AD being the cause in approximately three out of four cases.^1^ Nowadays, biomarkers such as amyloid-β (Aβ) or neurofibrillary tangles (tau) can detect AD years before clinical manifestation.^2,3^ This provides a window of opportunity to prevent or slow down disease progression prior to the onset of dementia.^4^ There is no one-size-fits-all approach to the diagnosis, prediction, and prevention of AD, as individuals differ in their preferences, needs, and wishes. Additionally, heterogeneity in terms of individual risk factor profiles and pathological pathways means that there will never be a single therapeutic strategy that works for all patients.^5–10^ In this vein, there is a need for personalization.

The concepts of ‘*personalized medicine*’ and ‘*patient-orchestrated care*’ are increasingly gaining attention in the AD field.^4,5,7,11–16^ While both concepts aim to customize care, care, their origins differ. The medical concept of *personalized medicine*, also referred to as precision medicine, stems from a biomedical framework aiming for optimized diagnostic, therapeutic, and preventive strategies based on an individual's genomic, epigenomic, and proteomic profile.^17,18^ The main goal of *personalized medicine* is to offer the ‘right drug for the right patient at the right time’.^19^ The field of *personalized medicine* incorporates the biopsychosocial model, focusing on the highly individual aspects of diagnosis and treatment while also considering their relevance to the patient.^20^ This may be achieved, for instance, via the use of individualized predictive modelling and frequent real-life data assessments.^20^ A former study identified 683 definitions of *personalized medicine*, which had 1449 different ends (i.e., aims) and 1025 different means (i.e., ways to reach the aim), mainly focusing on biomedical aspects (e.g., drug approval) as well as touching upon psychosocial factors (e.g., quality of life).^21^ Alternative to *personalized medicine*, the term *patient-orchestrated care* (or patient-centered/-directed/-focused care) originates from the 1940s-1950s and has since gained increasing prominence and formal recognition.^22–24^ *Patient-orchestrated care* represents a shift in focus from the traditional paternalistic biomedical model viewing individuals as passive targets of healthcare and the ‘doctor knows best’ approach, towards a model that prioritizes respecting personhood via maximizing individual autonomy and choice for those receiving healthcare.^23,25–28^ *Patient-orchestrated care* refers to care in which a person is involved and in control of decisions surrounding their own health and healthcare, for example by engaging the person in need of care, their knowledge (e.g., behavioral, biological, social aspects), and their care partners (i.e., loved ones, informal caregivers) in shared decision-making.^25,29,30^ In *patient-orchestrated care*, the values and preferences of the individual person inform all aspects of care via the individuals’ elicitation of voice.^31^ Healthcare professionals must design healthcare systems that are tailored to meet the individual needs of each person, rather than expecting individuals to adjust their needs to fit the existing healthcare system.^23,26–28^

*Personalized medicine* and *patient-orchestrated care* have sparked interest in the clinical context of AD due to recent advancements such as lifestyle interventions and disease-modifying treatment.^32,33^ Today, AD is no longer defined via a purely clinical diagnosis, but rather via the presence of biomarkers, which in turn allows for an AD diagnosis for people in pre-dementia stages.^3^ In combination with other recent biomedical advances allowing for early diagnosis, dementia risk prediction, and dementia prevention for people in pre-dementia stages, these recent advancements come with ethical challenges, such as whether clinicians should share an AD diagnosis with a patient before the onset of dementia.^34,35^ As a result, individuals in need of care, their care partners, and clinicians must make increasingly complex decisions. These decisions involve not only biomedical factors but also personal preferences, as they weigh the pros and cons of various options. For instance, decisions related to what people find important to be able to do in their daily lives or decisions related to what people consider to be a meaningful effect regarding recent disease-modifying treatments and what treatment burden and risk they are willing to accept.^36^ However, despite increased interest in *personalized medicine* and *patient-orchestrated care*, it is currently unclear how such terms are actually interpreted by professionals working in the AD field.

The conceptual vagueness of both *personalized medicine* and *patient-orchestrated care* complicate their implementation.^21,37–39^ Relatedly, it is important to address the conceptual vagueness by separating *personalized medicine* from patient-orchestrated care due to their different scopes and ethical considerations. Moreover, the challenges in the AD and broader dementia field are rendered wicked problems (i.e., problems that are difficult to solve due to their numerous interrelated components and diverse perspectives of stakeholders, such as health disparities, health-related stigma, long waiting lists in mental healthcare, or integrating information across healthcare systems),^40,41^ which can only be solved by involving the perspectives of multiple disciplines. Conceptual vagueness can create challenges in interdisciplinary collaboration, as a shared ‘language’ is key for effective communication and successful collaborations.^42^ This study, therefore, aimed to explore how AD professionals—engaged in interdisciplinary work—interpret the concepts of *personalized medicine* and *patient orchestrated care* and their considerations regarding the implementation of these concepts in the care trajectory.

## Methods

### Study design and context

This study adopted an interpretivist paradigm, acknowledging that knowledge is socially constructed and subjectively understood.^43^ We utilized a phenomenological methodology, conducting semi-structured interviews to explore the interpretations of professionals.^44–46^ In terms of context, this study was conducted as part of the ABOARD project. ABOARD is an acronym which stands for A *Personalized Medicine* Approach for Alzheimer's Disease, aiming to ensure a future of personalized, patient-orchestrated diagnoses, and the prediction and prevention of AD.^47^ The project's mission is to facilitate the implementation of *personalized medicine* for AD through the development of effective patient-orchestrated, predictive, diagnostic, and preventative strategies. It is a large-scale research project carried out by a public-private consortium in the Netherlands, comprising a surplus of thirty partners with a broad variety of relevant professions and backgrounds, namely ranging from healthcare, societal organizations (including patient advocacy organizations), industry, and academia and education. The project also has an end-user committee posed to offer advice. This committee includes members extending to family caregivers, clinicians, policy makers, implementation and valorization experts.

### Study sample, data saturation, and ethics

All senior-level professionals who were part of the ABOARD consortium (N = 42; senior meaning having a PhD, being a specialized clinician, or having senior management position) were invited for the study by e-mail in a random stepwise manner to minimize the risk of affinity bias (e.g., including participants similar to the researchers or those who we expect to have affinity with the topic). One professional declined participation and eleven professionals did not respond. This resulted in a final sample of thirty participating professionals that varied in professions (i.e., academia and education, clinicians, societal organizations, and ‘the industry’).

We specifically included participants from the industry because, as mentioned earlier, the field of AD is grappling with complex, ‘wicked’ problems that can only be addressed by incorporating perspectives from multiple disciplines. In this study, ‘industry’ refers to a range of organizations, including a brain health monitoring company, a biomarker assay company, a pharmaceutical company, and a healthcare consultancy firm. Clinicians are defined as healthcare professionals working in a memory clinic, such as physicians, nurses, and psychologists. As we had a heterogeneous participant group, where six to twelve participants is usually enough to reach data saturation, we expected the number of 30 participating professionals to be sufficient in achieving data saturation.^48^ Data saturation was checked for, and achieved, when no new themes seemed to occur. Participating professionals signed an informed consent form prior to the interview and were given a random identification code to maximize anonymity (indicated as ID-xxx). Professionals did not receive compensation for their participation in the study. The Medical Ethics Committee of the Amsterdam UMC, location AMC, declared that this study (W21_562 # 22.003) is not subject to the Dutch Medical Research Involving Human Subjects Act (WMO). This study complies with ethical standards of the Helsinki Declaration.^49^

### Interviews

The interviews were semi-structured using open-ended questions and follow-up questions.^50^ Semi-structured interviews are commonly used in health research and help explore the thoughts, feelings, and beliefs participants have about particular topics.^51^

Preliminary interview questions were developed based on the research aim and refined after extensive discussions within the research team. The questions were then pilot-tested with two clinicians working in a memory clinic, during which we sought feedback on the clarity and comprehensiveness of the questions. The interviews were pilot tested in January 2022 and subsequently conducted by TR (PhD student within the ABOARD project; trained in qualitative research). At the time of data collection, a pre-established relationship between TR and three ABOARD participants (10% of participants) had been identified as TR previously collaborated with one and worked with two that work within the project itself. Interviews were conducted in Spring 2022, either in-person at a location that was chosen by the participant, or online via Microsoft Teams. To ensure confidentiality, provide a comfortable environment, and minimize distractions, only the individual participant and the interviewer were present during the interviews. After personal introductions and an explanation on the reasons behind this study, the interviews started with an open-ended question exploring the concept of *personalized medicine* to capture their initial, key associations. For this, professionals were instructed to write down associations with *personalized medicine* within one minute on a post-it (live interviews) or word document (online interviews). In order to capture their interpretations and perspectives on the implementation of the terms *personalized medicine* and *patient-orchestrated care*, an open-ended 4 step question structure was conducted: (1) their interpretations of *personalized medicine* in AD, (2) their understanding of *patient-orchestrated care* in AD, (3) their view on the relationship between *personalized medicine* and *patient-orchestrated care*, and (4) their perception of the role of care partners in *personalized medicine* and *patient-orchestrated care* (for interview guide see supplementary materials). All with the goal to capture their interpretations and perspectives on implementation of the terms *personalized medicine* and *patient-orchestrated care*. The interviews were audio-recorded and lasted between 17 and 38 min. The average duration for in-person interviews was 28.6 min, while the average duration for online interviews was 28.2 min.

### Data analysis

All interviews were transcribed verbatim. After transcription, each participant received their responses to the four main questions for member checking, transcript reviewing, and interpretive validity. This is a qualitative research technique used to validate, verify, and assess the trustworthiness of the results.^52^ During the member check, we asked professionals if they agreed on the transcript, our interpretation of the summary and/or key statements of the interview, and whether they wanted to add or change anything. Five professionals provided additional responses, while twenty-five professionals agreed to the transcript as it was. The transcribed interviews were then analyzed by both DV (junior researcher; trained in qualitative research) and TR to reduce subjectivity via inductive coding using MAXQDA 2022.^53^ During analysis, DV could only see the identification code and did not know the name of the participant. Inductive coding is a method in which a researcher reads and interprets raw data with the aim of developing concepts, themes, or a model.^54^ Inductive coding differs from deductive coding as inductive coding is directly based on the data itself instead of based on prior assumptions, theories, or hypotheses.^54,55^ The written responses, in the form of a picture of a post-it or word document, were also included in the analysis. For the analysis, elements of interest were first identified by selecting coding units from the transcripts and written responses, i.e., the smallest parts that can make sense on their own.^56^ For this study, the coding unit was an element of a sentence describing a unique component of either *personalized medicine* or *patient-orchestrated care*. We first categorized each identified element of interest as part of *personalized medicine* and/or part of *patient-orchestrated care*. We used these elements of interest to generate illustrative word clouds on *personalized medicine* and *patient-orchestrated care* (see Results). Afterwards, we conducted a round of inductive coding to further subcategorize these elements using in vivo coding, axial coding, and subcoding.^53^ All interviews were coded independently by two researchers (DV and TR), after which they discussed results until a consensus was reached. The final coding scheme can be found in Supplemental Table 1. Next, thematic content analysis was used to identify patterns of meaning (themes) in the defined codes and subcodes,^57^ allowing for an overview of the interpretations and perspectives on implementation of professionals regarding the two concepts (see key statements in the result section).^57^ Finally, professionals were divided into ‘working as clinician’ versus ‘working exclusively in academia, education, societal organizations, or industry’ and checked thematically if any differences occurred. We did not conduct a full comparative analysis between clinicians and other participants due to the small sample size of participants primarily working as clinicians. In comparative analysis, sufficient representation within each group is necessary to draw meaningful conclusions. For reporting, we used the COnsolidated criteria for REporting Qualitative research (COREQ) guidelines.^58,59^

## Results

In total, thirty professionals were interviewed: nine (30%) working in healthcare, five (16.7%) in industry, six (20%) in societal organizations, and ten (33.3%) in academia and education. Their ages ranged from 28 to 63 years old (mean age: 46 years) and sixteen (53%) of the professionals were female.

### Personalized medicine

**Figure 1.**
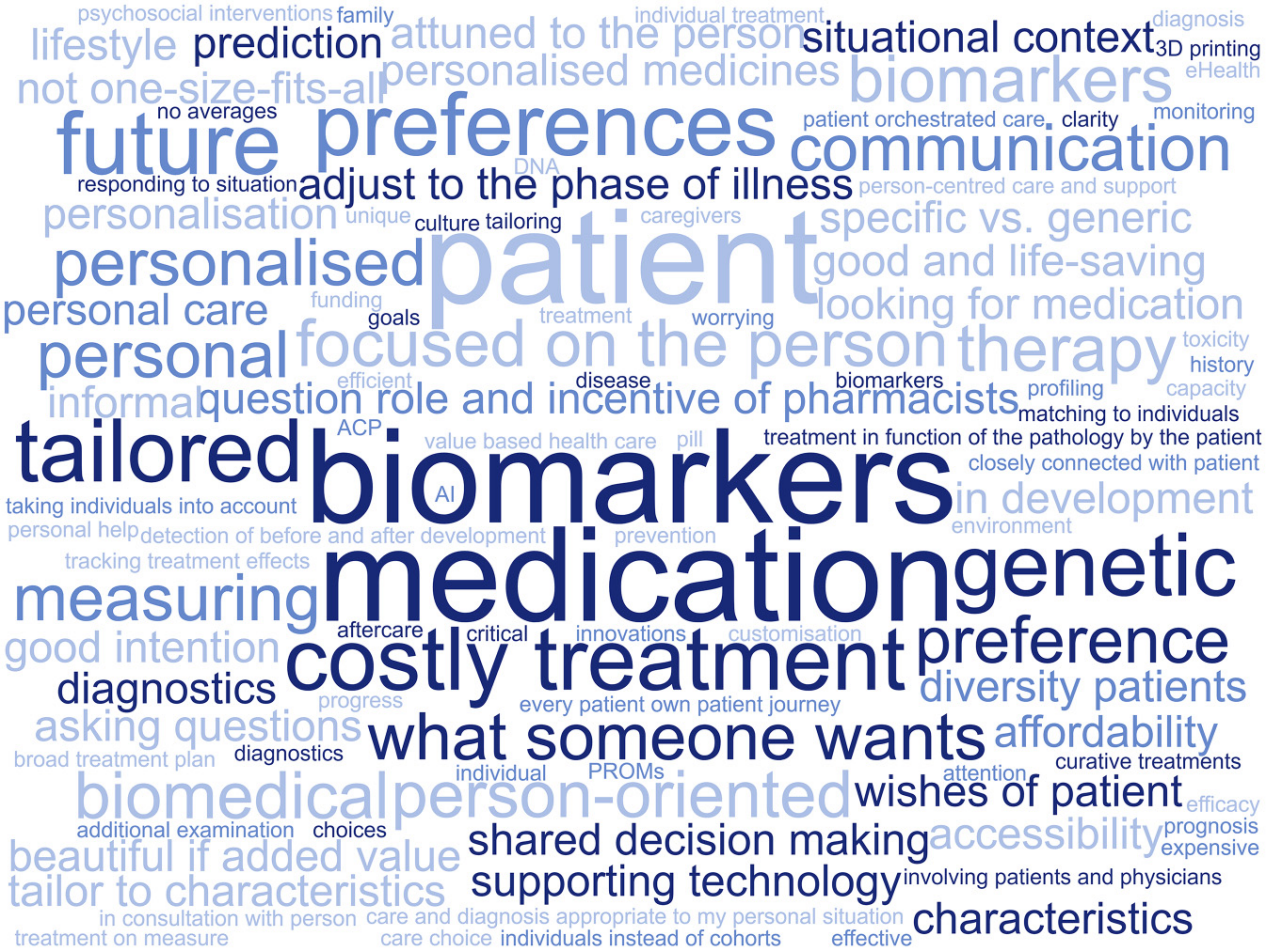
Words commonly associated with *personalized medicine* by professionals. Bigger words indicate words that are mentioned more often.

Professionals consider *personalized medicine* to be a biomedical approach as well as an approach which incorporates personal preferences, aimed at providing customized treatment. Figure 1 consists of key words that professionals associated with the term ‘*personalized medicine*’. This figure illustrates how professionals considered *personalized medicine* to be a biomedical approach as well as an approach that incorporates personal characteristics (i.e., age) and preferences (defined as a greater liking of something over another, e.g., making care-related decisions based on what a good quality of life is for someone). According to professionals, the aim to of *personalized medicine *is to provide customized treatment.

People receiving the right care, at the right moment, with the right treatment goal, in the right context for them as a person is what echoed through the interviews considering *personalized medicine*.

‘So indeed, what treatment, but also when and in what way then, in what place then, does that connect optimally? So not only in content but also in form and in timing’ (ID-461, psychologist & researcher, academia & education).

Treatment in the context of *personalized medicine* was understood by professionals as being informed both by biomedical characteristics and personal preferences of those in need of care. When discussing the role of biomedical characteristics in *personalized medicine*, professionals emphasized the tailoring of care, including treatment, to an individual's biological make-up and pharmacokinetics.

‘So personalized medicine to me is treatment according to the pathology of the patient. So, is the pathology present or not and is the treatment going to have an effect on the pathology? That to me is personalized medicine and then I think mainly about efficiency.’ (ID-455, CEO, industry).

When mentioning psychosocial factors as an element of *personalized medicine*, professionals referred to exploring personal preferences and the entire personal situation of a person in need of care. According to professionals, understanding an individual's situation, needs, and preferences is salient not only for reaching an accurate diagnosis, but also for determining the appropriate care goals, timing, and context. This insight enables clinicians to deliver personalized care that is coherently aligned with the unique needs of each individual. When discussing *personalized medicine* and the AD patient journey, professionals regarded AD and dementia as particularly suited for *personalized medicine* due to the heterogeneity of the underlying pathological processes.

Professionals differed in one aspect of how they interpreted *personalized medicine*: some professionals aimed for truly individualizing care by combining various biomedical and psychosocial characteristics from a single person (see quote below), while other professionals were more focused on establishing the best care options based on group level characteristics.

‘It is very difficult to come up with one approach, one treatment or one protocol that satisfies everybody. Because I believe that there is a lot of variability in what people want to know. How much diagnostic testing they want, how much certainty they want. That is… it wouldn't be different for everyone but there are a lot of variations, and you cannot predict it in advance based on general characteristics or something. So, the individual approach I think is very important with everyone, because otherwise it won't go well’ (ID-465, medical doctor, healthcare).

### Patient-orchestrated care

**Figure 2.**
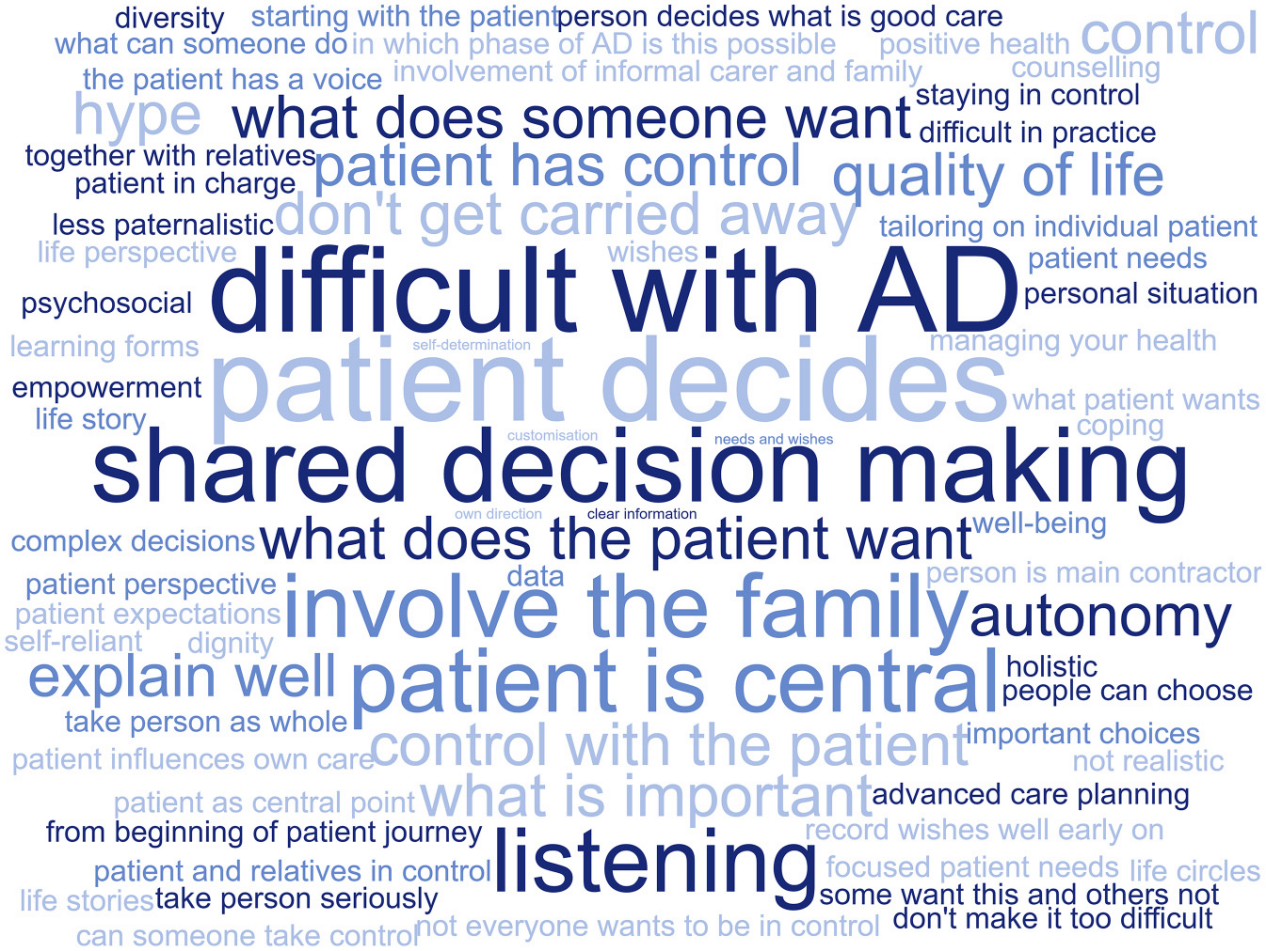
Words commonly associated with *patient-orchestrated care* by professionals. Bigger words indicate words that are mentioned more often.

*‘Patient-orchestrated care*’ is perceived by professionals as being similar to shared decision-making, involving the person with cognitive complaints, care partner, and the clinician in the decision-making process. Shared decision-making was considered as central in *patient-orchestrated care* (see Figure 2). Professionals indicated the importance of involving the person with cognitive complaints, care partner, and the clinician in the decision-making process in *patient-orchestrated care*. As professionals emphasize, the decision-making process should prioritize what the person in need of care values and considers important (see Figure 2). For *patient-orchestrated care* to happen, professionals mention that the person in need of care should thus be actively involved in care decisions and should be in control, to a certain degree.

‘And what is realistically possible, so that you, yes, that as a doctor you actually have the task of explaining and discussing all options in every step of the journey, that the patient can choose in consultation with their family. And the same actually applies to the whole patient journey.’ (ID-457, medical doctor, healthcare)

Two-way communication between the person in need of care and their clinician was another reported prerequisite for *patient-orchestrated care* by professionals: the individual should receive enough information to be able to take control, whereas the clinician should (get to) know the person's needs and preferences regarding the desirability of being in control (‘control preference’). *Patient-orchestrated care* was argued to take place throughout the entire AD patient journey, sometimes even before people visit the memory clinic, for instance, at home or at the general practitioner. However, professionals considered it to occur specifically when decisions regarding diagnostic testing and/or treatment must be made.

The clinician is responsible for aligning all parties involved and plays a coordinating role. Professionals highlighted that clinicians must regularly assess the level of control the person in need of care wants and can manage. Based on this assessment, clinicians should ideally empower the person to self-direct. However, in situations such as decisional conflicts or when the person does not want to take control, a more directive role may be necessary. Moreover, in case of disease progression, professionals mentioned that the clinician could take over (some of the) control together with the care partner. *Patient-orchestrated care* would then require clinicians to initiate timely conversations on who would have decisional responsibility when a person in need of care would be unable to express their own needs and preferences, for instance in the form of advanced care planning.

‘You have to look very much at what stage something can or cannot be done. With people with dementia, there is a tendency to think that they can no longer do this. At the same time, I also see a great overestimation of the extent to which people can take control of their own lives’ (ID-454, researcher, academia & education).

‘If a patient can oversee it [the situation] just fine and is happy to make the choice, of course that's fine too, but if it gets very complicated, the patient may well say, yes, doctor, that's what I am hiring you for. Tell me what is best for me, and I will follow. And then you have to go and do that too, so you cannot say “yes, you have to choose anyway” (ID-465, medical doctor, healthcare).

### Many professionals are in favor of applying patient-orchestrated AD care, whereas some professionals were hesitant

Professionals differed in their views on *patient-orchestrated care* regarding its desirability and feasibility. Many professionals found *patient-orchestrated care* for AD desirable, whereas the other professionals were more hesitant. Professionals who were in favor of *patient-orchestrated care* linked it to positive outcomes, such as an increased quality of life, dignity throughout the patient journey, and increased feelings of control and empowerment for the person in need of care. These professionals explained that clinicians may not always be as skilled as patients in evaluating decision options, as the process is deeply influenced by the individual's values and priorities. Additionally, they viewed *patient-orchestrated care* as a timely approach, especially with the emergence of Alzheimer's medications, which carry both potential benefits and risks, alongside the growing trend of individual autonomy in society.

'So that also requires that the patient themselves actually weighs off, which is very difficult for that patient themself, but are the side effects worth it versus the duration or quality of life that you can still gain. And that is not a purely technical consideration, but that has to do with what you find important in your life’ (ID-441, health insurer, societal organization).

The professionals who were more hesitant regarding *patient-orchestrated care* questioned the level of control people should have, i.e., how much responsibility an individual person should have about their own care. Some had doubts regarding patient safety; for instance, they feared that individuals would turn to ‘quack doctors’ if patients were given full control over their own care. Other professionals questioned whether individuals actually want to take the lead in their care. They wondered if *patient-orchestrated care* is both feasible and desirable if the person does not wish to be in control. Concerns were raised that this approach could place an additional burden on individuals, leading to more decisions to be made, which could become overwhelming. These professionals frequently mentioned that *patient-orchestrated care* is not appropriate for everyone, since: a) not everyone wants to be in control, b) AD is a progressive condition, potentially making it harder for people to be in control due to disease progression, c) decisions that have to be made are very complex, and d) insight is needed in all the medical and care possibilities to really participate.

‘That the citizen is in control of their own [care], to determine what the best care and support is for him or her. I want to make the important note that it is very difficult for someone to grasp the full range of support possibilities. It is also challenging to comprehend that.’ (ID-446, researcher, academia & education)

### Overlap between personalized medicine and *patient-orchestrated care*

There is overlap between *personalized medicine* and *patient-orchestrated care*, where three themes notably emerged. Firstly, as discussed previously, professionals recommended to take personal preferences, wishes, values, and situation of the person in need of care into account for both *personalized medicine* and *patient-orchestrated care*. Secondly, most professionals believed that clinicians are responsible for achieving *personalized medicine* by providing available personalized treatment options, but also for *patient-orchestrated care,* by coordinating the process and providing relevant information. Thirdly, implementation challenges persist for both *personalized medicine* and *patient-orchestrated care*, subsequently elaborated on.

### Personalized medicine and *patient-orchestrated care* are both considered part of the patient journey, yet professionals also see challenges regarding the implementation of these terms in clinical practice

Although *personalized medicine* and *patient-orchestrated care* are both considered part of the patient journey (of the future), professionals also saw challenges regarding their implementation in clinical practice. Especially in the light of emerging, but still limited, therapeutic options, the relevance and timeliness of *personalized medicine* and *patient-orchestrated care* was stressed. While some professionals argued that effective, approved therapeutic options for AD are a prerequisite for the implementation of *personalized medicine*, other professionals countered this idea.

‘But I think prognostic and diagnostic care is actually irrelevant if you cannot treat. So, then you don't need to go to the doctor either, so I think all the diagnostic and prognostic stuff is fine. But if that doesn't lead to you being able to treat someone then I actually think it's a bit of a shame. After all, that's in nobody's interest’ (ID-447, researcher, healthcare).

‘So, I see that very often people think yes it [prognostic and diagnostic care] doesn't really matter all that much because you can't do anything about it anyway. So why pursue it at all … I find it quite fatalistic’ (ID-456, medical doctor & researcher, healthcare).

Professionals agreed that it is a challenge to implement *personalized medicine* and *patient-orchestrated care* in AD healthcare practice. One professional suggested that eHealth could support the implementation of *patient-orchestrated care*. However, other professionals argued that the healthcare system is not yet prepared, highlighting the need for a shift in both the mindset of professionals and the current practices in memory clinics.

‘Customization sounds great. Everyone just wants to be seen or heard and treated as an individual. But it is also a trade-off when something is of such added value that it pays off, so to speak, and when it is a nice ambition, but the effort it requires is not justified’ (ID-441, health insurer, societal organization).

‘To make it work in practice, and I think that does require a different way of thinking. And also, a different way of acting in practice. And a different relationship between the professional, the patient and the informal carer. And it should not be underestimated what it takes to get that done’ (ID-461, psychologist & researcher, academia & education).

Some professionals debated whether *personalized medicine* should focus solely on the person in need of care or also include their care partners. Concerns were raised about the potential compromise of the person's autonomy, particularly in cases where decisional conflicts arise between the person affected and their care partner. This was especially concerned when a care partner, having assumed responsibility, acts out of self-interest rather than prioritizing the individual's needs.

'So if one person has somewhat different ideas than the other, that does create some difficulty sometimes to do that [decision making] very much from a patient preference. Because from which preference do you act exactly? Who is leading then, that also often presents professionals with difficulties of how to deal with that properly’ (ID-461, psychologist & researcher, academia & education).

‘We have also experienced from previous discussions with informal caregivers that sometimes there are differences in how an informal caregiver interprets things and perhaps thinks things [the medical situation] are better compared to how the patient experiences it. So, there is a certain area of tension when you talk about personalized medicine and autonomy. Yes, the informal caregiver plays a very important role, but I think you have to constantly evaluate or determine or probe what is the best match for the patient's wishes, just like in the interaction between patients and caregiver’ (ID-446, researcher, academia & education).

### Differences between clinicians and other Ad-related professionals

Although in the analyses we specifically looked for differences between clinicians and other AD-related professionals in terms of their interpretations and perspectives on implementation, we could not thematically identify any differences.

## Discussion

In this study, we explored the interpretations of, and perspectives on, the implementation of a broad range of professionals engaged in interdisciplinary work in the AD field regarding the concepts *personalized medicine* and *patient-orchestrated care*. We found that, according to professionals, *personalized medicine* permits relevant options for diagnosis and disease management informed by biomedical as well as psychosocial factors. In contrast, *patient-orchestrated care* captures factors relevant to the decision-making process, such as who is involved and the involvement of others in care decisions at different stages of the disease trajectory. Professionals generally agreed in their views on *personalized medicine* but had broader interpretations of, and differed in, their views on *patient-orchestrated care* regarding its desirability and feasibility. *Personalized medicine* and *patient-orchestrated care* have some overlap according to the professionals since both terms incorporate patient preferences and consider the clinician to be the facilitator and ultimately responsible. Taken together, implementation challenges for both concepts remain.

Professionals had similar views and perspectives on initial operationalization's of *personalized medicine*, reflecting shared understanding and vision. Personalized medicine has been around for several years in other medical specialties, such as oncology, and is (currently being) implemented more broadly in the healthcare field as well.^60–63^ Previous research in the AD field showed some benefits to applying *personalized medicine*. For instance, the diagnostic accuracy of AD has been improved, particularly after incorporating biomarkers as diagnostic criteria.^7,64^ This is in line with findings from our study in the sense that biomarkers and genetics were also often referred to throughout the interviews as part of a *personalized medicine* approach. A similar study on *personalized medicine* in the chronic inflammation setting found that clinicians and researchers foresee several potential benefits, including more efficient patient selection for clinical trials, more cost-effective treatment pathways, increased therapy response, and reduced side-effects.^17,65^ Participants also anticipated several ethical, procedural, and economic challenges, including the potential risks of data misuse and discrimination, the possibility that certain groups may not have access to therapy, the need for ongoing professional support, and the necessity of political measures to promote healthy lifestyles. Additional concerns included difficulties some populations may face in accessing inflammation clinics and the financial challenges of making care accessible to all.^65^ The potential benefits mentioned are in line with results from our study, in which professionals acknowledge the potential of *personalized medicine* for increased therapy response and reduced side effects. The economic aspect regarding *personalized medicine* was brought up in our study. However, cost-effectiveness was discussed more as a prerequisite of and in relation to the desirability of *personalized medicine*, which touches upon the challenge of financing *personalized medicine* and the accessibility of healthcare. Other challenges commonly discussed in the chronic inflammation setting were not raised by professionals in our study. This may indicate that those in the AD field are currently unaware of these issues. A critical discussion of these challenges would be advisable before moving forward with implementation.

What was surprising in the interview segments on *personalized medicine* was that professionals explicitly mentioned personal preferences as being part of *personalized medicine* for AD, which is currently not reflected in the AD literature.^7,14–16,66,67^ In the specific context of AD, personal preferences in *personalized medicine* are accounted for in some studies, such as an economic evaluation study,^68^ some touching upon patient engagement,^69,70^ or a study on the perception of *personalized medicine* in the general practitioner (GP) setting.^17^ The latter study also explored professionals’ perspectives on *personalized medicine* determining that many GPs interpret *personalized medicine* as an approach that regards the whole person, including their physical, psychological, and social characteristics.^17^ *Personalized medicine* in the GP setting is understood as taking a global view of each patient's unique situation, beyond genetics, and heredity.^17^ However, the incorporation of personal preferences is not reflected in all of the conceptual studies on *personalized medicine*.^71,72^ This absence of personal preferences in *personalized medicine* is highlighted by a review, which revealed few studies incorporating patients’ individual needs, beliefs, behavior, values, wishes, utilities, environment, and/or circumstances in definitions on *personalized medicine* compared to physiology-based approaches.^73^ Other studies, mostly in oncology, acknowledge the potential of incorporating shared decision-making and taking preferences into account in *personalized medicine*, but also show that the implementation hereof is not clearly established yet,^74–76^ thus, highlighting an implementation gap.

Professionals in our study considered the process of shared decision-making as key to* patient-orchestrated care*. Shared decision-making is ‘*an approach where clinicians and patients share the best available evidence when faced with the task of making decisions, and where patients are supported to consider options, to achieve informed preferences’.*^77^ Previous research shows that shared decision-making is associated with increased satisfaction, increased trust, higher quality of life, patient empowerment, and reduction in anxiety and other negative emotions.^78,79^ In the context of AD, we know from previous research that people in need of care and care partners would like to be actively involved in decisions around diagnostic testing for AD.^80^ Professionals in the current study also emphasized that people in need of care should be actively involved, for instance because clinicians may not be as adept as people themselves at evaluating different options when making decisions, considering that the decision process is inherently tied to individual values and priorities in life. Still, some professionals stressed that *patient-orchestrated care* may not be suitable for every person in need of care, highlighting the need of tailoring to the specific situation and the preferences and capabilities of the individual.

From a physician perspective, engaging in shared decision-making in the AD field can be challenging. Previous research showed that half of patients and care partners currently do not discuss what they expect and would like to achieve at the memory clinic with their physicians. This makes it harder for physicians to engage patients in shared decision-making.^81^ Also, previous research highlighted that there is room for improvement regarding shared decision-making in the AD field.^80,82,83^ Shared decision-making might be difficult to achieve in the AD context because *patient-orchestrated care* is considered more like triadic decision-making, whereby the care partner is involved.^84^ This brings challenges such as assessing someone's decisional capacity or addressing different consult expectations and information needs between a person in need of care and care partner.^84^ Furthermore, previous studies have indicated a discrepancy between the perceptions of values and quality of life of persons in need of care, particularly among those in mid or advanced stages of dementia, and their care partners.^85–89^ This may also contribute to challenges in decision-making within the triadic context. This emphasizes the necessity for clinicians to engage in early discussions with individuals requiring care and their care partners, and to monitor and assess this potential discrepancy over time. Memory clinic clinicians indicated that they would like to receive support regarding communication with patients, for instance including shared decision-making and triadic communication.^90^ In our data, it also seems that professionals who are more hesitant of *patient-orchestrated care* misunderstand shared decision-making as a process in which an extensive amount or even full control is being given to the person in need of care, which is for instance reflected in their worry of people not being able to get an overview of the available options, their fear of people turning to quack doctors, or the worry of forcing people to decide even though they do not want to or are able to. This is in line with commonly mentioned doubts surrounding shared decision-making.^91^ Moreover, some professionals mentioned that engaging in shared decision-making costs too much consultation time, however, this has been debunked in research.^92^ These challenges and lack of consensus on shared decision-making as part of *patient-orchestrated care* highlight the need for further research and discussions on the concepts and its ideal operationalization in the AD field.

In broader literature, professional's perspectives on *patient-orchestrated care* are described in different settings. Professionals working in a hospital setting understand key elements of *patient-orchestrated care* as treating patients with dignity and respect, an interdisciplinary approach, and having equal access and good outcomes.^93^ In an ambulatory care settings, *patient-orchestrated care* is mainly understood by professionals as having dignity and respect towards the patient, including building relationships, providing individualized care, and respecting patients’ time.^94^ Aligned with our results, professionals and patients alike view *patient-orchestrated care* in end-stage renal disease as involving several key elements: actively listening to patients and considering their treatment preferences, providing clear information and education to empower patients to take charge of their own care, fostering a supportive atmosphere in the department (including the physical environment, ambiance, interpersonal dynamics, and emotional climate), and having a designated care coordinator, either a professional or acquaintance, who works with the patient to make care decisions.^95^ These results are in line with our results. What differs from our results are the explicit mentions of waiting times, the atmosphere at the department, and a direct focus on health equity and accessibility.

Other future directions emerging from this study include the need to address the implementation challenges identified by professionals for *personalized medicine* and patient-orchestrated care. Further implementation research should incorporate personal, inter-personal, and systemic level implementation facilitators.^96^ Personal level implementation facilitators, for instance, include motivations and attitudes on *personalized medicine* and *patient-orchestrated care* in the AD context.^96^ Inter-personal level implementation facilitators comprises showcasing best practices, addressing the broader social network and influence, and involving patients.^96^ Systemic level implementation facilitators for instance include facilitating healthcare policies.^96^ These multi-level implementation changes require resources and support from the entire AD field, such as clinicians, health insurers, creators of medical guidelines, and policy makers. Furthermore, stakeholders ought to be involved closely from the beginning since implementation of *personalized medicine* and *patient-orchestrated care* can convincingly better fit the needs, values, and preferences of patients, which may increase the quality of AD care.

A strength of this study is that we included professionals from across the quadruple helix, including professionals from healthcare, industry, societal organization, academia and education to allow for an overview that is assumed to be representative for the entire AD field. Also, the semi-structured interviews allowed us to get an in-depth, bottom-up insight on the interpretations and perspectives on implementation of *personalized medicine* and *patient-orchestrated care* in the AD field. One could argue recruiting only professionals from the ABOARD project may limit the generalizability of findings from this study, however, qualitative research stems from interpretivism, which prioritizes comprehending human behavior over prediction, and extrapolating causes and effects.^97^ Generalizability of findings in qualitative studies is therefore in general not relevant nor a measure of quality, rather, having an in-depth understanding of situations and actions is.^98–102^ This is facilitated in our study via our choice for conducting semi-structured interviews, which help to explore participant thoughts, feelings, and beliefs about a particular topic.^51^ This study also has some limitations worth mentioning. The ABOARD project incorporates *personalized medicine* and *patient-orchestrated care* in its aims. Professionals within the ABOARD project are therewith more likely to know about these concepts and as such have a relatively positive attitude towards such concepts and be early adopters of them. Recruitment of professionals from the ABOARD project thus caused a selection bias. Moreover, the ABOARD project focuses on the patient journey starting after referral by the general practitioner until the diagnosis at the memory clinic, thereby excluding the patient journey stages before visiting the memory clinic and after receiving a diagnosis. Consequently, professionals included in this study focused on those stages during the interviews, which can for instance be seen by the fact that ‘diagnosis’ appeared as a key theme and by memory clinic doctors frequently being mentioned as a main actor in both *personalized medicine* and *patient-orchestrated care*. This leaves gaps regarding the interpretations and perspectives on implementation in other AD-related settings, such as consults with a general practice nurse or general practitioner, nursing home care, and residential care. The interpretations, perspectives on implementation, and the possibilities of *personalized medicine* and *patient-orchestrated care* in these settings may be different from findings in our study. Due to pragmatic reasons, we only included senior-level professionals in the study; however, additional or contrasting perspectives may exist among junior-level professionals, people in need of care, and care partners, which were not captured. These factors indicate potential areas of further exploration. Additionally, the lack of field notes during or after the interviews may have limited the depth of our data set.

### Conclusion

Mapping the interpretations, and perspectives on implementation, of *personalized medicine* and *patient-orchestrated care* in the field of AD is important given the recent developments in the AD field allowing for early diagnosis, dementia risk prediction, dementia prevention, and emerging disease-modifying treatments. These developments highlight the need for a care model in which individuals have control over their own care, while unnecessary interventions are minimized. *Personalized medicine* and *patient-orchestrated care* are thus seen as part of AD care today and in the near future. A shared understanding, interpretations, and vision seem to exist for *personalized medicine* across AD professionals, explicitly incorporating patient-preferences, which is not yet reflected in common definitions on *personalized medicine* within and beyond the AD field. *Patient-orchestrated care* requires further consensus and discussions on its meaning and implications, but touches upon key elements in common definitions, such as shared decision-making. Implementation facilitators and barriers for incorporating *personalized medicine* and *patient-orchestrated care* in AD-related care need to be addressed.

## Supplemental Material

sj-docx-1-alz-10.1177_13872877251326166 - Supplemental material for Exploring interdisciplinary perspectives on the implementation of *personalized medicine* and *patient-orchestrated care* in Alzheimer's disease: A qualitative study within the ABOARD research project Supplemental material, sj-docx-1-alz-10.1177_13872877251326166 for Exploring interdisciplinary perspectives on the implementation of *personalized medicine* and *patient-orchestrated care* in Alzheimer's disease: A qualitative study within the ABOARD research project by Tanja J de Rijke, Dianne Vasseur, Wiesje M van der Flier, Mirella MN Minkman, Hanneke FM Rhodius-Meester, Nicolaas A Verwey, Ellen MA Smets and Leonie NC Visser in Journal of Alzheimer's Disease
