# Supplementary material for: Exploring interdisciplinary perspectives on the implementation of personalized medicine and patient-orchestrated care in Alzheimer's disease: A qualitative study within the ABOARD research project
Source: J Alzheimers Dis. 2025 Mar 21;105(1):120–33. doi: 10.1177/13872877251326166 (PMC12231872; doi:10.1177/13872877251326166)
Supplement: sj-docx-1-alz-10.1177_13872877251326166 - Supplemental material for Exploring interdisciplinary perspectives on the implementation of personalized medicine and patient-orchestrated care in Alzheimer's disease: A qualitative study within the ABOARD research project [file sj-docx-1-alz-10.1177_13872877251326166.docx]

**Supplemental Material**

**Exploring interdisciplinary perspectives on the implementation of personalized medicine and patient-orchestrated care in Alzheimer’s disease: A qualitative study within the ABOARD research project**

**Supplemental Table 1. Overview of all codes derived bottom-up from data set.**

| **Top-level code** | **Subcodes** |
| --- | --- |
| Psychosocial | - |
| Individual client in network | - |
| Terminology | 1. A lot of English terminology (what is it about precisely; creates distance) 2. Opinions differ 3. Essential 4. Empty promise 5. Counselling 6. Hype term/buzz term 7. Container term 8. Personal 9. Personalised 10. PM is part of POC 11. It is close 12. unique |
| Detecting progress and decline | 1. measuring non-invasively |
| Research | - |
| Technical/technological/ biomedical approach | 1. eHealth 2. Medical part is only a small part of care 3. Subordinate to control with person |
| Quality of life/wellbeing | 1. Caring for instead of making sure that 2. Positive health |
| Prevention/lifestyle | 1. Behaviour change 2. Get a better grip |
| Patient-centred approach | 1. Patient journey (who comes to who; starts with diagnosis; starts at the general practitioner; start to think about control early on) 2. Persona’s (accessible environment) |
| Value-based healthcare | - |
| Continue to monitor development critically | - |
| Efficiency | 1. Healthcare system is in the way of patient-centred care 2. Balance efficiency versus applicability |
| Costs | 1. Cost-efficiency 2. More tests needed 3. Accessibility of healthcare is under pressure 4. Affordability of the healthcare system is under pressure |
| Technology supporting | - |
| Future | 1. Patient journey is changing 2. Innovation (system innovation in healthcare) 3. Under development (education; research; step in between) 4. Meagre and sad 5. Empty promise |
| Customisation | 1. At the right place 2. (not) considering characteristics (genes; literacy; disease type; disease phase; age; ethnicity; gender) 3. Desirable versus technically best solution 4. (not) different with AD 5. Customisation based on organisation and care form 6. Tailored care 7. At the right moment 8. Goals 9. Tailored 10. Balance customisation versus generic (on individual level) |
| Role of healthcare professionals | 1. Taking control in case of conflict between person and care partner 2. Taking control if person themselves is unable to 3. Thinking in solutions (not in problems) & seeing the bigger picture 4. Responsibility properly inform what is important to someone 5. Giving advice based on knowledge that matches someone's values 6. Tailor care based on wishes and needs 7. Outline framework/offer grip 8. Diagnosis guidance 9. Bonding/build trust 10. Coaching role 11. Initiative from healthcare professionals 12. Providing care based on supply is central 13. Healthcare professional does not have the central role 14. Change of perspective 15. Offer care control based on need 16. Giving a choice to think about the condition 17. Less paternalistic 18. Giving space for a person to take control 19. Focus on self-reliance and daily life applications (control with the person or healthcare professional?) |
| Treatment | 1. Flexibility is important 2. Starts at home 3. Aftercare 4. Aimed at cure 5. Healthcare makes no sense if there is no treatment 6. Early on 7. Learning styles 8. Self-learning |
| Medication | 1. Under development (alternatives needed now) 2. Much to be gained from personalised medicine |
| Care partners | 1. Consequences of decisions for care partners 2. Wishes of care partners 3. Personalisation for care partners 4. Under/overestimation of situation 5. Deserve attention (psychological support) 6. How does this work for care partners? 7. Are affected by disease 8. Relief and support of care partners |
| Role of care partners | 1. Care partner will become end-user as well 2. Support and care/care partner (role in treatment; to stay at home) 3. Involve from the beginning 4. Dilemma (decisional conflict between person and care partner; autonomy may be compromised; what if care partner does not take control?; best intentions?; increasingly the voice of person; personalisation with or without informal carer) 5. Control with the person  control with care partner 6. Changes over time 7. Involve closely with patient journey 8. Reciprocal relationship 9. Information source of a person 10. Important role (sometimes affected more by the disease; will become conscience of the person; will become working memory of person; more important as dependency increases with AD) |
| Control with person | 1. Having a voice in development of care 2. Choice must be possible 3. Autonomy 4. Not suitable for everyone (no insight in options; intelligence; doctor knows best/do not get carried away; how much control does someone want; limited disease insight; depending on disease phase) 5. (feeling of) empowerment 6. Prepare course of direction 7. In practice hard to implement 8. What may a person do themselves and what not? 9. Customisation may restrict control with the person (responsibility is the downside of control; complexity) 10. Conditional 11. A requirement for patient-orchestrated care 12. Better judgment than doctor 13. Self-managing 14. Self-personalisation/orchestrating 15. Saying no 16. (not) deciding for yourself/making care decisions 17. Having in a voice in the organisation of care (starts at home; may start to bite with care; does not mean that it will always go that way) 18. Shared decision-making (important with AD; weighing options is difficult for person; with healthcare professional; with care partner and healthcare professional; with care partner) 19. Informed decision making |
| Decisions based on data | 1. Artificial intelligence 2. Decisions based on your own data 3. Patient reported outcome measures 4. Detecting patterns in stories 5. Learning from groups (unpredictable; not suitable for personal preferences) 6. Subgroups 7. Prognosis 8. Prediction (risk stratification) 9. Not based on n=1 10. Sketching from a broader perspective |
| Social network | 1. Coordination with other people 2. Care load capacity 3. Coordination of healthcare professionals 4. Requires coordination 5. Mismatch care partners current situation |
| Person | 1. Undertakes interaction 2. Determining for the choices that you make 3. Person-centred 4. Starting point |
| Diagnosis | 1. Early diagnosis 2. Additional examinations based on differential diagnosis 3. Who and how should people be involved 4. What is the impact on someone’s life 5. Clear what the condition is 6. What are the wishes 7. How to cope with diagnosis 8. Realising what the diagnosis means |
| Communication | 1. Family situation 2. Living situation 3. Considering: (who must be involved (diagnosis); (information) need; age) 4. Not a lot of reflection on actions 5. By mutual agreement 6. Who and how should people be involved 7. Miscommunication (between person and care partner) 8. What are the wishes 9. What is the impact on someone’s life 10. Clarity 11. Clarity on what the diagnosis is 12. How to cope with diagnosis 13. Realising what the diagnosis is 14. Feedback 15. Taking seriously 16. Involve in information exchange 17. Explanation (pros and cons of different options; costs time; continuously loop back to this; from patient perspective) 18. Treatment 19. Asking questions 20. Understandable language |
| Delving into personal situation | 1. May change over time 2. Preferences 3. Goals 4. Perspective 5. Context (living environment; living situation; family situation) 6. What matches target group perception 7. Culture 8. Who and how should be involved 9. What is the impact on someone’s life 10. Clear what condition is 11. How to cope with diagnosis 12. Who should be there 13. Especially for the first time 14. Fear for future 15. Coping 16. How are you today 17. Health 18. Care 19. Diagnosis 20. Request for help 21. Talking about it 22. What suits someone 23. Care load capacity 24. Life story 25. How does someone perceive life 26. Life in dignity 27. Wishes of a person 28. What is important in life 29. Needs of care partners 30. Needs of person 31. More important if you cannot do anything else |

**Supplemental Material: Interview guide**

1. For this part of the interview, I would like to ask you to write down all your associations, thoughts, or feelings on a post-it regarding the theme ‘’personalised medicine’’. You have one minute to write down as many thoughts, associations, or feelings regarding this theme.
2. What is your view on personalised medicine in AD?
3. What is your understanding of the term ‘patient-orchestrated care’ in AD?
4. How does this term [POC] relate to personalised medicine from your perspective?
5. How do you see the role of care partners in personalised medicine and patient-orchestrated care?
6. Do you have any additional questions or additions regarding the themes that we discussed?
